# Attenuation of Adaptive Thermogenesis by a Culinary Dose of Red Pepper During 24-Hour Negative Energy Balance

**DOI:** 10.1093/nutrit/nuaf300

**Published:** 2026-05-26

**Authors:** Mary-Jon Ludy, George E Moore, Richard D Mattes, Margriet S Westerterp-Plantenga

**Affiliations:** Graduate College, Bowling Green State University, Bowling Green, OH, 43403, United States; Department of Veterinary Administration, Purdue University, West Lafayette, IN, 47907, United States; Department of Nutrition Science, Department of Public Health, Purdue University, West Lafayette, IN, 47907, United States; Department of Nutrition and Movement Sciences, Maastricht University, Maastricht, MD, 6200, The Netherlands

**Keywords:** capsaicin, red pepper, energy balance, adaptive thermogenesis, energy intake, energy expenditure, fat oxidation, hunger suppression, weight maintenance, diet-induced thermogenesis, satiety

## Abstract

Obesity treatment requires weight loss through a negative energy balance (EB) followed by sustained weight maintenance. Adaptive thermogenesis, increased hunger, and decreased fullness challenge long-term success. Capsaicin, the pungent principle in red pepper (RP), has been reported to modestly increase energy expenditure (EE) and fat oxidation (lower respiratory quotient [RQ]) while reducing energy intake (EI). This article updates a previous review and meta-analyses (1995–2010), incorporating studies published through 2024, to evaluate whether culinary RP doses can attenuate adaptive thermogenesis and decreased fullness during negative EB. Twenty-two publications representing 24 randomized controlled studies were synthesized. Meta-analyses assessed EE and RQ differentiated by duration, dose, EB status, and protein level. One-meal studies showed that EE (diet-induced thermogenesis) and RQ changes did not reach statistical significance. Long-term studies revealed that EE changes did not reach statistical significance, whereas RQ was consistently lower, indicating enhanced fat oxidation. Appetite and EI findings were mixed. Designs were heterogenous and possibly too short to capture sustained effects or too long to ensure adherence. To address these limitations, we incorporated 24-hour controlled respiratory chamber studies comparing capsaicin and capsaicin combined with elevated protein vs placebo across all 3 meals, in both neutral and negative EB. Meta-analyses showed that EE increases during negative EB did not reach statistical significance overall, although individual studies suggest that capsaicin may modestly increase EE compared with placebo. Capsaicin significantly reduced RQ in negative-EB and in high-protein conditions, indicating greater fat oxidation. Capsaicin also counteracted decreased fullness during negative EB. Culinary RP doses do not fully prevent adaptive thermogenesis but may attenuate its impact during negative EB, particularly when paired with protein. Enhanced fat oxidation and improved fullness indicate potential benefits for weight-loss maintenance. Long-term EE effects remain minimal, but sustained RQ reductions support capsaicin as an adjunct to dietary weight-loss maintenance strategies.

## INTRODUCTION

Obesity is a worldwide chronic disease that often leads to comorbidities such as type 2 diabetes, cardiometabolic issues, and metabolic syndrome.^1^ Effective treatment requires weight loss through a long-term negative energy balance (EB) followed by sustained weight maintenance.^2–4^ Achieving a negative EB can be accomplished through lifestyle interventions, pharmacotherapy, and/or bariatric surgery.^2–4^ Lifestyle interventions encompass reducing energy intake (EI), sustaining energy expenditure (EE) while sparing fat-free mass (FFM), and maintaining circadian alignment.^5^ Unfortunately, weight maintenance is often short-lived due to weight regain.^6^ A reduction in body weight and changes in body composition decrease EE and thus energy requirements.^5^ This decrease in EE is often larger than expected based on achieved body weight and body composition, a phenomenon known as adaptive thermogenesis or metabolic adaptation.^7–10^ Despite the decrease in energy requirements, maintaining a lower EI is difficult due to appetitive factors such as hunger and desire to eat, previous eating habits, and the rewarding value of food.^9^^,^^11^

Relatively higher protein diets, which reduce total EI by lowering fat and carbohydrate intake, have shown benefits such as sustained satiety, preserved FFM, and sustained EE that counteract adaptive thermogenesis. These diets also promote weight loss and maintenance of the achieved weight for up to 2 years.^11–14^ However, relying on a single food ingredient may not be sufficient to fulfill all requirements for successful weight management. A mix of food ingredients, including those with thermogenic properties, such as red pepper (RP) with capsaicin as its pungent principle and limited energy itself, may play a role in preventing adaptive thermogenesis.^15^^,^^16^ Capsaicin, signaled through the transient receptor potential vanilloid 1 (TRPV1) receptor, induces thermogenesis by β-adrenergic stimulation.^17–19^ It has been reported to increase sympathetic nervous system activity, fat oxidation, and insulin synthesis; reduce adipocyte size; and promote the conversion of white adipose tissue to brown adipose tissue.^20–22^

With regard to appetite (**Table 1**), the ingestion of capsaicin vs placebo with a meal has been reported to decrease hunger and desire to eat, increase fullness and satiety, and reduce EI,^24^^,^^26^^,^^32^^,^^33^^,^^37^^,^^39–41^ although other studies observed no effect.^23^^,^^25^^,^^27^^,^^28^^,^^42^^,^^44^ Satiety effects of capsaicin may be related to altered taste perception,^45–47^ which can alter neural responses to tastants via taste receptor cells, both independently of and dependent upon trigeminal transmission.^48–53^

**Table 1.** Studies on the Effects of Capsaicin on Thermogenesis and Appetite Updated From Ludy et al^19^: 1-Meal, Low-Dose Studies—Energy Balance

Study (year)	Participants	Experimental design/intervention	Thermogenesis	Appetite
Rigamonti et al (2018)^23^	6 ♂ and 4 ♀ in Italy Age 21.0 ± 5.8 y BMI 41.5 ± 4.3 kg/m^2^ (mean ± SD)	Single-blind randomized crossover (2 visits) with pre-lunch capsule containing: 1. 2 mg capsaicin (*Capsicum annum*) 2. Placebo	↑ REE	No effect on hunger, satiety, ghrelin, GLP-1, or PYY No effect on EI at an ad libitum dinner
van Avesaat et al (2016)^24^	5 ♂ and 7 ♀ in the Netherlands Age 21.5 ± 0.6 y BMI 22.8 ± 0.6 kg/m^2^ (mean ± SE)	Single-blind randomized crossover (2 visits) with intraduodenal infusion over 30 min containing: 1. 1.5 mg pure capsaicin 2. Placebo (physiologic saline)	n/a	↑ Satiety No effect on hunger, GLP-1, or PYY
Swint et al (2015)^a^^,25^	11 ♂ and 13 ♀ in the USA (Ohio) Age 25.2 ± 10.2 y BMI 21.7 ± 2.4 kg/m^2^ 8 regular spicy-food users, 13 weekly-to-monthly users, 3 nonusers (mean ± SD)	Single-blind randomized crossover (3 visits) with lunch containing: 1. 1 g cayenne RP (2 mg capsaicin) 2. 1 g CH-19 sweet pepper (2 mg capsiate) 3. Placebo Ad libitum dinner	n/a	No effect on fullness, hunger, desire to eat, urge to eat, preoccupation with food, thirst, or cravings for something salty, fatty, or sweet No effect on EI at an ad libitum dinner
Ludy and Mattes (2011)^a^^,26^	14 ♂ and 11 ♀ in the USA (Indiana) Age 23.0 ± 0.5 y BMI 22.6 ± 0.3 kg/m^2^ 13 regular spicy-food users, 12 nonusers (mean ± SE)	Randomized crossover (6 visits) with lunch containing cayenne RP (1995 μg/g capsaicin, 53 800 SHU) or placebo: 1. 1 g RP (following ↑ FAT diet) 2. 1 g RP (following ↑ CHO diet) 3. Placebo (following ↑ FAT diet) 4. Placebo (following ↑ CHO diet) 5. Preferred RP dose—oral (1.8 ± 0.3 g users; 0.3 ± 0.1 g nonusers) 6. Preferred dose RP dose—capsule (1.8 ± 0.3 g users; 0.3 ± 0.1 g nonusers)	↑ EE, core body temperature, and fat oxidation (when consumed orally compared with in capsule form) ↓ Skin temperature	↓ Preoccupation with food and desire to eat fatty/salty/sweet foods in nonusers but not users ↓ EI in nonusers, but not users No effect on desire to eat in general, fullness, prospective food intake, thirst, or hunger
Reinbach et al (2010)^27^	17 ♂ and 23 ♀ in Denmark Age 24.6 ± 2.5 y BMI 22.5 ± 7 kg/m^2^ Likers of hot spices (mean ± SD)	Randomized crossover (10 visits) with “starter meal”: 1. With or without chili peppers (0.3 g RP; 0.375 mg capsaicin) 2. With or without ginger 3. With or without mustard 4. With or without horseradish 5. With or without wasabi Ad libitum dinner	n/a	↑ Desire to eat sweet foods ↓ Desire to eat hot foods No effect on EI, food intake (g), hunger, satiety, or desire to eat bitter/fatty/salty/sour (results for RP only)
Smeets and Westerterp-Plantenga (2009)^28^	11 ♂ and 19 ♀ in the Netherlands Age 31 ± 14 y BMI 23.8 ± 2.8 kg/m^2^ (mean ± SD)	Randomized crossover (2 visits), single-blind with lunch containing: 1. 1.03 g cayenne RP; 80 000 SHU 2. Placebo (without RP)	No effect on DIT or RQ	↑ GLP-1 15 min after capsaicin lunch Ghrelin tended to ↓ 15 min after capsaicin lunch (*P* = .07) No effect on PYY or satiety
Chaiyata et al (2003)^29^	12 ♀ in Thailand Consumers of <10 g/d chili peppers	Glucose drink containing: 5 g fresh chili pepper (*Capsicum frutescens*): 3.5 mg capsaicin	↑ EE above RMR immediately after ingestion	n/a
Matsumoto et al (2000)^30^	8 lean ♀ in Japan Age 19.6 ± 0.26 y BMI 21.0 ± 0.57 kg/m^2^ 8 overweight/obese ♀ in Japan Age 20.1 ± 0.40 y BMI 28.8 ± 1.01 kg/m^2^ No long-term history of eating spices (mean ± SE)	Randomized crossover (2 visits) with breakfast containing: 1. 3 mg capsaicin 2. Placebo (without capsaicin)	↑ EE in lean but not overweight/obese individuals ↑ Total frequency, very low-frequency, low-frequency, and the ratio of very low- to total-frequency heart waves (SNS activity) in lean but not overweight/obese individuals Trend toward ↑ CHO oxidation in lean but no effect in entire group No effect on ↑ frequency heart waves (PSNS activity) in either group	n/a

a Reflects funding from the McCormick Science Institute.

Abbreviations: BMI, body mass index, CHO, carbohydrate; DIT, diet-induced thermogenesis; EB, energy balance; EE, energy expenditure; EI, energy intake; FAT, fat; GLP-1, glucagon-like peptide 1; n/a, not applicable; PSNS, parasympathetic nervous system; PRO, protein; PYY, peptide YY; RP, red pepper; REE, resting energy expenditure; RMR, resting metabolic rate; SHU, Scoville Heat Unit; SNS, sympathetic nervous system; VAS, visual analog scale; ♀, females; ♂, males.

A previous review and meta-analyses on the effects of culinary doses of RP on thermogenic and appetitive outcomes in humans demonstrated proof of concept. Capsaicin was found to increase EE and fat oxidation, particularly at high doses, although results on the possible reduction in EI were mixed.^19^ In the present narrative review and updated meta-analysis, we evaluated whether culinary doses of RP can attenuate adaptive thermogenesis and decreased fullness during negative EB in humans. First, the previous review and meta-analysis, based on articles published from 1995 to 2010, was updated to include studies published through 2024. Additionally, we assessed whether effects differed according to duration, dose, EB status, and protein level. Since 1-meal studies may have been too brief to capture sustained effects and long-term studies too extended to ensure participant adherence, we incorporated controlled 24-hour studies to address these gaps. These studies were designed to test whether capsaicin, alone or combined with elevated protein across all 3 meals, could offset reductions in EE and fullness typically observed during negative EB, under controlled respiratory chamber conditions.

## METHODS

To build upon the previous review and meta-analyses,^19^ we conducted a comprehensive literature search using PubMed and Google Scholar. Key words included RP, capsaicin, EE, energy metabolism, diet-induced thermogenesis (DIT), thermogenesis, fat oxidation, respiratory quotient (RQ), substrate oxidation, hunger, satiety, fullness, desire to eat, food intake, EI, and humans. The search was limited to English-language publications from 1995 through 2024. Eligible studies were experimental in design, conducted in humans, and reported outcomes related to EE, substrate oxidation, and/or appetite following capsaicin ingestion. Studies were excluded if they involved animals, lacked a placebo/control condition, or did not report a capsaicin dose. Unpublished data were excluded; however, 1 included study was a published master’s thesis.^31^

This search yielded 7 new publications:^23–25^^,^^31^^,^^37–39^ Two focused exclusively on thermogenesis,^31^^,^^38^ 3 focused exclusively on appetite,^24^^,^^25^^,^^37^ and 2 addressed both thermogenesis and appetite.^23^^,^^39^ In total, 22 studies were included in the review.

**Figure 1.**
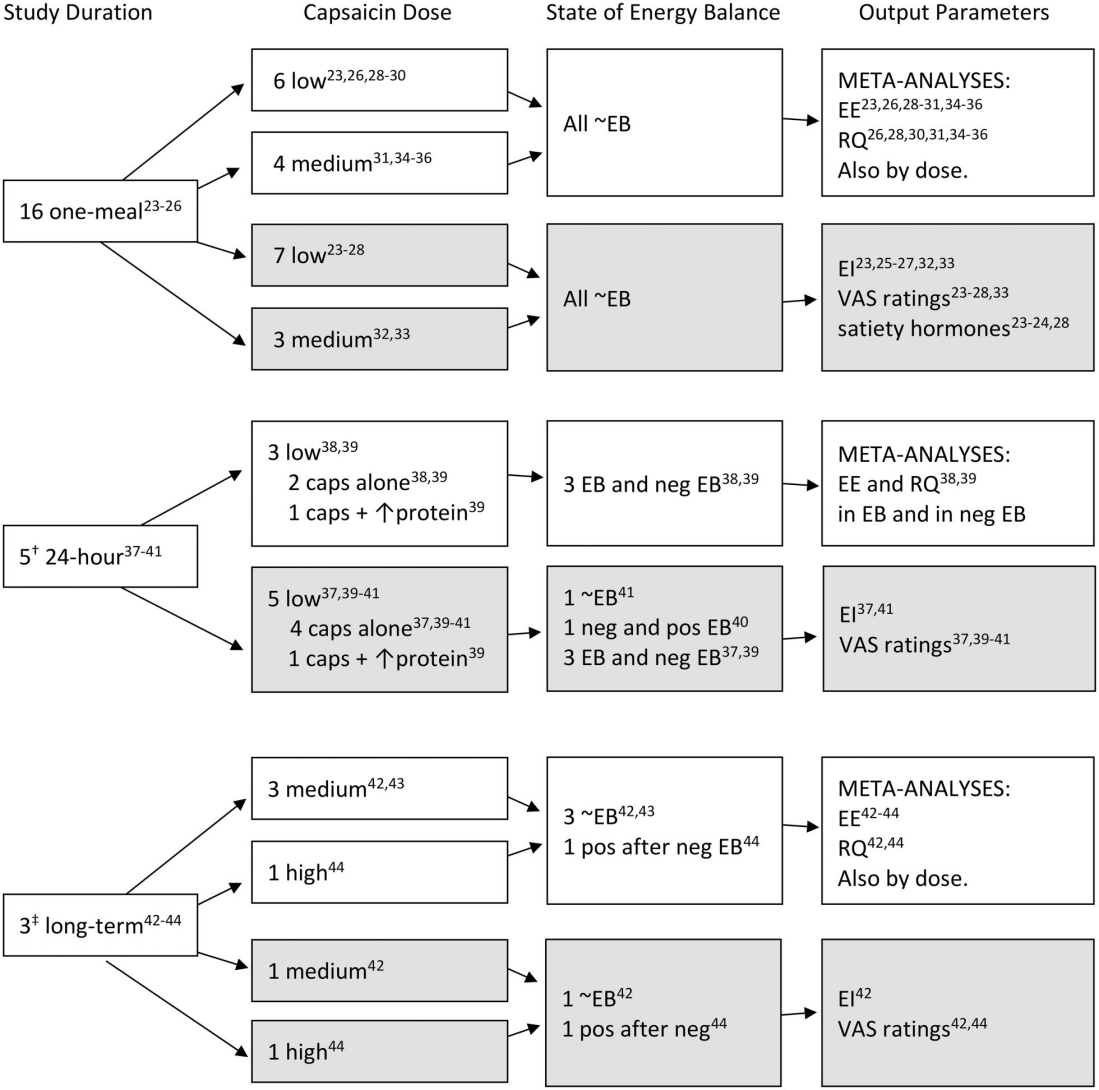
Overview of 22 Publications Including 24 Studies Included in the Review and Meta-analysis Publications are categorized by study duration (1-meal, 24-hour, long-term), capsaicin (caps) dose (low: 0.2–7.68 mg/meal; medium: 20.4–33 mg/meal; high: 135 mg/d over 3 meals), and state of energy balance (EB) (neutral: ∼100% energy requirements met; negative [neg]: 75%–80% energy requirements met; positive [pos]: during weight regain after a period of negative EB). Output parameters include energy expenditure (EE) and respiratory quotient (RQ) for thermogenesis (white boxes), as well as energy intake (EI), self-perceived ratings via visual analog scale (VAS), and satiety hormones for appetite (gray boxes). ^†^Smeets et al^39^ included multiple arms: one with capsaicin alone and another combining capsaicin with high protein. ^‡^Ahuja et al^43^ also included multiple arms: one following a 4-week chili diet and another following a 4-week bland diet.

Studies were categorized by duration (1-meal, 24-hour, or long-term), dose (low: 0.2–7.68 mg/meal: medium: 20.4–33 mg/meal; high: 135 mg/d across 3 meals), and EB (neutral, negative, or positive) (see **Figure 1**). Dose categories were adapted from our previous meta-analysis,^19^ which defined low doses as 7 mg or less of capsaicin, medium doses as 20–35 mg, and high doses as 135–150 mg. These cutoffs reflect the distribution of doses in available studies and practical considerations, including exposure frequency and sociocultural norms that influence spicy food preference and tolerance.^54^ Low doses are typical of Western diets, where spicy food consumption is modest and preferred concentrations average approximately 0.6 and 3.6 mg per meal among nonusers and regular users, respectively.^26^ Medium doses are more characteristic of Asian diets, where higher spice levels are customary and meals may contain up to approximately 30 mg capsaicin.^34–35^ High doses exceed culinary norms and are achievable only through encapsulated supplements, such as those used in long-term studies providing 135 mg per day.^44^

**Tables 1–4** present studies by duration, dose, and EB status: 1-meal, low-dose studies in EB (**Table 1**)^23–30^; 1-meal, medium-dose studies in EB (**Table 2**)^31–36^; 24-hour, low-dose studies in EB, negative EB, and positive EB (**Table 3**)^37–41^; and long-term studies including medium doses in EB^42^^,^^43^ and a high dose in positive EB following weight loss (**Table 4**).^44^ Two studies are listed twice due to distinct participant groups (ie, Japanese women vs White men in Yoshioka et al^33^) or mechanistic interventions (ie, β-adrenergic blockers in Yoshioka et al^36^).

**Table 2.** Studies on the Effects of Capsaicin on Thermogenesis and Appetite Updated From Ludy et al^19^: 1-Meal, Medium-Dose Studies—Energy Balance

Study (year)	Participants	Experimental design/intervention	Thermogenesis	Appetite
Virk (2019)^31^	4 ♂ and 6 ♀ in the USA (Virginia) Age 29 ± 6 y BMI 28.2 ± 3.5 kg/m^2^ (mean ± SD)	Crossover (2 visits): Day 1: Placebo Day 2: 8.2 g cayenne RP (20.4 mg capsaicin)	↑ Fat oxidation ↑ EE	n/a
Yoshioka et al (2004)^32^	16 ♂ in Japan Age 22.4 ± 3.2 y Wt 79.4 ± 19.4 kg Ht 176.1 ± 6.7 cm (mean ± SD)	Randomized crossover with pre-lunch soup containing: 1. Self-perceived moderate RP soup (0.064 ± 0.046 g; 0.192 ± 0.138 mg capsaicin; 55 000 SHU) and capsule (placebo) 2. Self-perceived strong RP soup (0.923 ± 1.377 g; 2.769 ± 4.131 mg capsaicin; 55 000 SHU) and capsule (placebo) 3. Soup (placebo) and self-perceived strong RP capsules (0.923 ± 1.377 g; 2.769 ± 4.131 mg capsaicin; 55 000 SHU) 4. Soup (placebo) and capsule (placebo) Ad libitum lunch until satiated	Biphasic effect on SNS: PSNS (moderate = maximum tolerable > strongest/excessive)	Dose-dependent ↓ EI (soup = capsules) *P *= .08 Dose-dependent ↓ fat intake (soup = capsules) Positive correlation between EI and fat intake Negative correlation between change in EI and RP ingested
Yoshioka et al (1999)^33^ Study 1	13 Japanese ♀ Age 25.8 ± 2.8 y Wt 54.2 ± 6.4 kg Ht 157 ± 4 cm Accustomed to eating spicy foods (mean ± SD)	Randomized crossover (4 visits) with breakfast containing: 1. 10 g RP (↑ FAT, 30 mg capsaicin) 2. 10 g RP (↑ CHO, 30 mg capsaicin) 3. 0 g RP (↑ FAT, placebo) 4. 0 g RP (↑ CHO, placebo) Ad libitum lunch buffet until satiated	n/a	↓ Lunch intake 1. PRO (↑ FAT > ↑ CHO) 2. FAT (↑ FAT > ↑ CHO) Self-reported appetitive sensations 1. Prospective food consumption ↓ before lunch, but ↑ after lunch 2. ↓ Desire to eat and hunger immediately after breakfast and before lunch
Yoshioka et al (1999)^33^ Study 2	10 White ♂ Age 32.9 ± 7.8 y Wt 72.5 ± 10.1 kg Ht 175 ± 6 cm (mean ± SD)	Randomized crossover (2 visits) with lunch appetizers containing: 1. 6 g RP (18 mg capsaicin) 2. Placebo (without capsaicin) Ad libitum lunch and snack buffet until satiated	↑ SNS: PSNS Trend toward a negative correlation between SNS: PSNS and EI	↓ Total energy and CHO intakes during lunch and snack
Yoshioka et al (1998)^34^	13 Japanese ♀ Age 25.8 ± 2.8 y Wt 54.2 ± 6.4 kg Ht 157.3 ± 4.5 cm Hotness of habitual meals between RP and placebo meals (mean ± SD)	Randomized crossover (4 visits) with breakfast containing: 1. 10 g RP (↑ FAT) 2. 10 g RP (↑ CHO) 3. 0 g RP (placebo, ↑ FAT) 4. 0 g RP (placebo, ↑ CHO)	↑ EE (VO_2_) and fat oxidation	↓ Appearance, taste, and smell ↑ Hot sensation
Lim et al (1997)^35^	8 ♂ Long-distance runners in Korea Age 20.8 ± 0.5 y Wt 58.5 ± 2.1 kg Ht 169.5 ± 1.8 cm (mean ± SE)	Randomized crossover (2 visits) with breakfast containing: 1. 10 g RP (30 mg capsaicin) 2. 0 g RP (placebo) Followed by 2.5 h rest, then 1 h cycling	↑ CHO oxidation both at rest and during exercise ↑ Epinephrine and norepinephrine 30 min after meal No effect on VO_2_	n/a
Yoshioka et al (1995)^36^ Study 1	8 ♂ Long-distance runners Age 20.5 ± 1 y Wt 58.5 ± 5.6 kg Ht 169.5 ± 4.7 cm (mean ± SD)	Randomized crossover (2 visits) with breakfast containing: 1. 10 g RP (30 mg capsaicin) 2. 0 g RP(placebo)	↑ CHO oxidation Tended to ↑ EE (VO_2_)	n/a
Yoshioka et al (1995)^36^ Study 2	7 ♂ Long-distance runners Age 21.6 ± 0.7 y Wt 61.3 ± 11.6 kg Ht 170.9 ± 6.3 cm (mean ± SD)	Randomized crossover (2 visits) with breakfast containing 10 g RP (30 mg capsaicin) followed by: 1. β-Adrenergic blocker (propranolol) 2. Placebo	Propranolol inhibited the initial ↑ in EE caused by RP	n/a

Abbreviations: BMI, body mass index, CHO, carbohydrate; EE, energy expenditure; EI, energy intake; FAT, fat; Ht, height; n/a, not applicable; PRO, protein; PSNS, parasympathetic nervous system; RP, red pepper; REE, resting energy expenditure; SHU, Scoville Heat Unit; SNS, sympathetic nervous system; VO_2_, oxygen consumption; Wt, weight; ♀, females; ♂, males.

**Table 3.** Studies on the Effects of Capsaicin on Thermogenesis and Appetite Updated From Ludy et al^19^: 24-Hour, Low-Dose Studies—Energy Balance, Negative Energy Balance, and Positive Energy Balance

**Study (year)**	Participants	Experimental design/intervention	Thermogenesis	Appetite
Janssens et al (2014)^a,37^	8 ♂ and 7 ♀ in the Netherlands Age 29.7 ± 10.8 y BMI 23.3 ± 2.9 kg/m^2^ Regular spicy-food users (≥ weekly) (mean ± SD)	Single-blind randomized crossover (4 visits) in EB and negative EB containing 7.68 mg capsaicin or placebo over 3 meals (3.09 g RP: *Capsicum frutescens L* and *Capsicum annuum L*; 39 050 SHU): 1. 100% EB capsaicin 2. 100% EB placebo 3. 75% EB capsaicin 4. 75% EB placebo	n/a	↑ Satiety and ↑ fullness in EB Capsaicin counteracted the effects of negative EB on after-dinner desire to eat, satiety, and fullness (ie, 75% EB capsaicin did not differ from 100% EB placebo) Tended to ↓ ad libitum EI and over-consumption in EB
Janssens et al (2013)^a,38^	8 ♂ and 7 ♀ in the Netherlands Age 29.7 ± 10.8 y BMI 23.3 ± 2.9 kg/m^2^ Regular spicy-food users (mean ± SD)	Single-blind randomized crossover (4 visits) in EB and negative EB containing 7.68 mg capsaicin or placebo over 3 meals (3.09 g RP: *Capsicum frutescens L* and *Capsicum annuum L*; 39 050 SHU): 1. 100% EB capsaicin 2. 100% EB placebo 3. 75% EB capsaicin 4. 75% EB placebo	Capsaicin counteracted the effects of negative EB on DIT and REE compared with placebo (ie, DIT and REE in 75% EB capsaicin did not differ from 100% EB placebo, while 75% EB placebo did ↓ compared to 100% EB placebo) ↑ Fat oxidation in negative EB	n/a
Smeets et al (2013)^39^	12 ♂ and 12 ♀ in the Netherlands Age 27 ± 4 y BMI 25.2 ± 0.4 kg/m^2^ (mean ± SD)	Single-blind randomized crossover (8 visits) in EB and negative EB containing 3.09 mg cayenne RP or placebo over 3 meals in 2 capsules (40 000 SHU) with normal or ↑ PRO (10% or 25% kcal, respectively): 1. 100% EB placebo 2. 80% EB placebo 3. 100% EB capsaicin 4. 80% EB capsaicin 5. 100% EB placebo with ↑ PRO 6. 80% EB placebo with ↑ PRO 7. 100% EB capsaicin with ↑ PRO 8. 80% EB capsaicin with ↑ PRO	↑ EE with capsaicin and PRO, both alone and mixed (ie, 100% EB capsaicin with ↑ PRO > 100% EB capsaicin = 100% EB placebo with ↑ PRO = 80% EB capsaicin with ↑ PRO > 80% EB placebo with ↑ PRO > 100% EB placebo = 80% EB capsaicin > 80% EB placebo) ↑ Fat oxidation with capsaicin and PRO, both alone and mixed (ie, 80% EB capsaicin with ↑ PRO > 80% EB capsaicin = 80% EB placebo with ↑ PRO = 100% EB capsaicin with ↑ PRO > 80% EB placebo = 100% EB placebo with ↑ PRO > 100% EB capsaicin > 100% EB placebo)	↑ Fullness with capsaicin and PRO, both alone and mixed (ie, 100% EB capsaicin with ↑ PRO > 100% EB placebo with ↑ PRO = 80% EB capsaicin with ↑ PRO > 80% EB placebo with ↑ PRO = 100% EB capsaicin > 80% EB capsaicin = 100% EB placebo > 80% EB placebo)
Reinbach et al (2009)^40^	10 ♂ and 17 ♀ in the Netherlands Age 26.9 ± 6.3 y BMI 22.2 ± 2.7 kg/m^2^ (mean ± SD)	Randomized crossover (10 visits) following 3-wk periods of negative and positive EB with 3 meals each containing: 1. Capsaicin capsules (0.51 g cayenne RP; 40 000 SHU) 2. Green tea drink (598.5 mg catechins, 77 mg caffeine) 3. CH-19 sweet pepper capsules (2.3 mg capsiate) 4. Green tea drink (598.5 mg catechins, 77 mg caffeine) + capsaicin capsules (0.51 g cayenne RP; 40 000 SHU) 5. Placebo capsules Ad libitum dinner	n/a	Capsaicin + green tea ↓ EI compared with placebo in positive (but not negative) EB, while capsaicin alone had no effect on EI Capsaicin + green tea ↓ hunger, ↓ desire to eat, ↑ fullness, and ↑ satiety more in negative than positive EB, while capsaicin alone ↓ desire to eat fatty, salty, hot, and sour stimuli in both positive and negative EB (results for capsaicin and capsaicin + green tea only)
Westerterp-Plantenga et al (2005)^41^	12 White ♂ and 12 White ♀ in the Netherlands Age 25–45 y BMI 25 ± 2.4 kg/m^2^ Used to eating spicy foods ≥ 1×/wk (mean ± SD)	Randomized crossover (4 visits) in EB with 2-d treatments 30 min before every meal containing RP (2.25 mg capsaicin, 80 000 SHU) or placebo: 1. 0.9 g RP in tomato juice 2. 0.9 g RP in 2 capsules 3. Placebo in tomato juice 4. Placebo in 2 capsules	n/a	↓ EI (juice > capsules) ↑ Satiety and CHO intake ↓ Hunger, energy density, and fat intake

a Reflects funding from the McCormick Science Institute.

Abbreviations: BMI, body mass index, CHO, carbohydrate; DIT, diet-induced thermogenesis; EB, energy balance; EE, energy expenditure; EI, energy intake; FAT, fat; n/a, not applicable; PRO, protein; RP, red pepper; REE, resting energy expenditure; SHU, Scoville Heat Unit; ♀, females; ♂, males.

**Table 4.** Studies on the Effects of Capsaicin on Thermogenesis and Appetite Updated From Ludy et al^19^: Long-Term Studies—Medium Dose in Energy Balance and High Dose in Positive Energy Balance Following Weight Loss

**Study (year)**	Participants	Experimental design/intervention	Thermogenesis	Appetite
Ahuja et al (2007)^42^	14 ♂ and 22 ♀ in Australia Age 46 ± 12 y BMI 26.3 ± 4.6 kg/m^2^ < Daily chili (∼ 90% naive/infrequent consumers) (mean ± SD)	Randomized crossover with 4-wk dietary periods containing: 1. Chili (30 g/d chili blend, 55% cayenne RP; 33 mg/d capsaicin) 2. Bland, spice free	No effect on RMR, RQ, fat oxidation, BMI, fat mass, or lean mass	No effect on total energy, PRO, FAT, or CHO intake
Ahuja et al (2006)^43^	14 ♂ and 22 ♀ in Australia Age 46 ± 12 y BMI 26.3 ± 4.6 kg/m^2^ < Daily chili (∼ 90% naive/infrequent consumers) (mean ± SD)	Randomized crossover with 4-wk dietary periods containing: 1. Chili (30 g/d chili blend, 55% cayenne RP; 33 mg/d capsaicin) 2. Bland, spice free	↓ EE BMI ≥26.3 kg/m^2^ No effect on EE using all BMIs or BMI <26.3 kg/m^2^	n/a
Lejeune et al (2003)^44^	91 Participants in the Netherlands Capsaicin: 12 ♂ and 30 ♀ Placebo: 11 ♂ and 38 ♀ Age 18–60 y BMI 25–35 kg/m^2^ Not habitual capsaicin users	Positive EB after negative EB, double-blind randomized 4-wk very-low-energy diet intervention (mean ↓ 6.6 ± 2 kg or 7.8 ± 1.8% body weight) followed by 3-mo weight-maintenance period with supplements containing: 1. 135 mg/d capsaicin (capsules with 3 meals) 2. Placebo (without capsaicin) (mean ± SD)	↑ REE during weight maintenance ↑ Fat oxidation No effect on percentage or rate of regain	No effect on hunger or satiety

Abbreviations: BMI, body mass index, CHO, carbohydrate; EB, energy balance; EE, energy expenditure; EI, energy intake; FAT, fat; n/a, not applicable; PRO, protein; RP, red pepper; REE, resting energy expenditure; RMR, resting metabolic rate; RQ, respiratory quotient.

Only studies reporting thermogenic parameters (EE and/or RQ) were included in the meta-analyses. Four studies contributed multiple arms to the meta-analyses due to differences in dose (ie, standard vs preferred dose in Ludy and Mattes ^26^), EB condition (ie, neutral vs negative EB in Smeets et al^39^ and Janssens et al^38^), and dietary patterns (ie, normal vs high protein in Smeets et al^39^ and spicy vs bland lead-in diet in Ahuja et al^43^) These distinctions allowed for more nuanced comparisons across dose, EB, and dietary pattern conditions.

Study quality and risk of bias were assessed as follows. For thermogenic studies, validated measurements such as indirect calorimetry were required. For appetite studies, 1-meal and 24-hour designs were required to be conducted in laboratory settings, with outcomes assessed via visual analog scales, blood samples, and/or measured EI from preweighed food. Participants were assumed to be approximately in EB, recognizing that true EB is typically achieved over 1 week due to daily variability.^55^

Meta-analyses on EE and substrate oxidation (RQ, calculated as expired CO_2_/inhaled O_2_) were performed within each duration category and according to dose, EB status, and protein level. The standardized mean difference (SMD) with 95% CI was used as an effect size indicator. Significant differences were indicated when the 95% CI did not include zero. *I^2^*, a measure of heterogeneity, was used to assess the proportion of inconsistency in individual studies not explained by chance. *I^2^* ranges between 0% and 100%, with lower values representing less heterogeneity. Nonsignificant *I^2^ P* values (ie, *P* > .05) indicate that heterogeneity was likely to occur by chance alone, suggesting that pooled data would not likely lead to bias. When heterogeneity was high, results were interpreted with caution and considered in the context of study design. Forest plots were generated with STATA SE version 18.5 (StataCorp, College Station, TX).

The impact of capsaicin vs placebo on appetitive sensations, ad libitum EI, and/or gut hormone concentrations (eg, ghrelin, glucagon-like peptide 1 [GLP-1], peptide YY [PYY]), or combinations thereof, is described within the same duration, dose, EB, and protein categories used for EE and substrate oxidation. Meta-analyses were not possible due to the diversity of the outcome parameters. All studies meeting inclusion criteria were considered in the narrative review. Emphasis was placed on studies with clearly reported outcome measures, but no formal weighting was applied.

To address the central research question—whether a culinary dose of RP consumed at each meal can attenuate adaptive thermogenesis and decreased fullness during negative EB—randomized controlled 24-hour crossover studies were emphasized. Three publications,^37–39^ all conducted since the previous review and meta-analyses, used respiratory chamber conditions to compare low-dose capsaicin with placebo across all 3 meals. Two publications^38^^,^^39^ reported thermogenic outcomes and were included in the meta-analyses. Each featured multiple arms comparing capsaicin’s effect under varying EB (neutral vs negative EB),^38^^,^^39^ and one also compared dietary conditions (normal vs high protein).^39^ The third publication,^37^ which also included neutral and negative EB conditions, reported only appetitive outcomes and was therefore included in the narrative synthesis but not in the meta-analyses.

## RESULTS

### Overall Effects on EE and RQ

**Figure 2.**
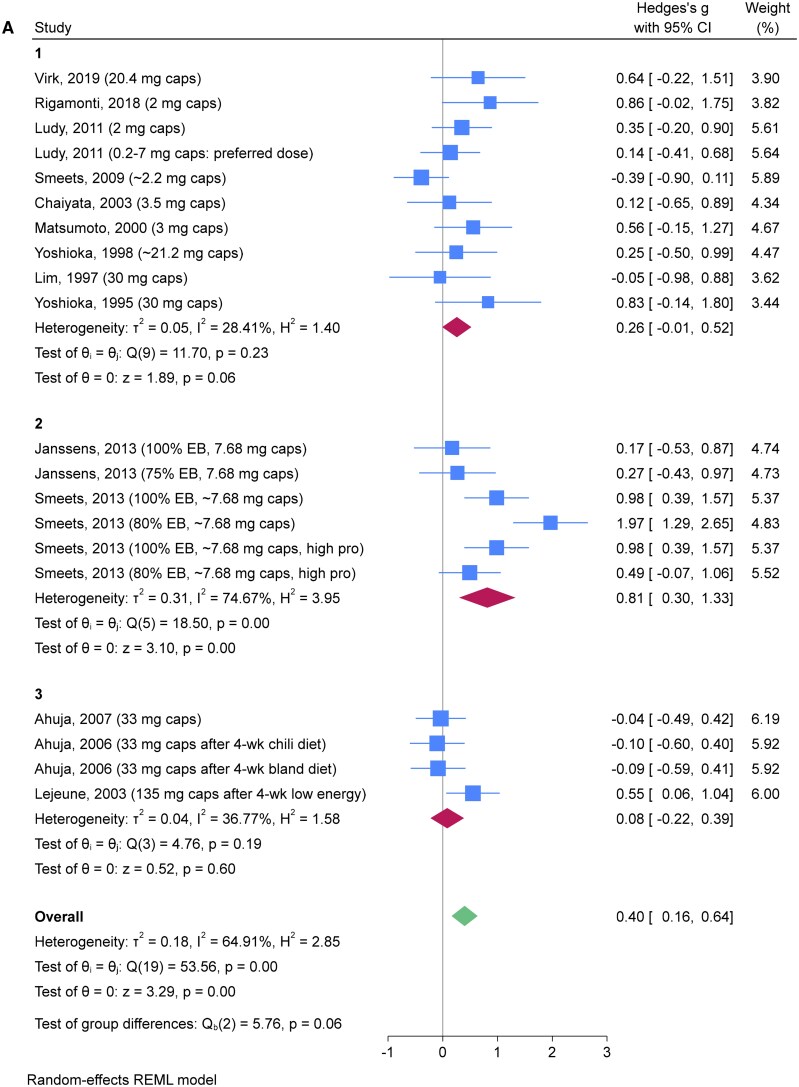

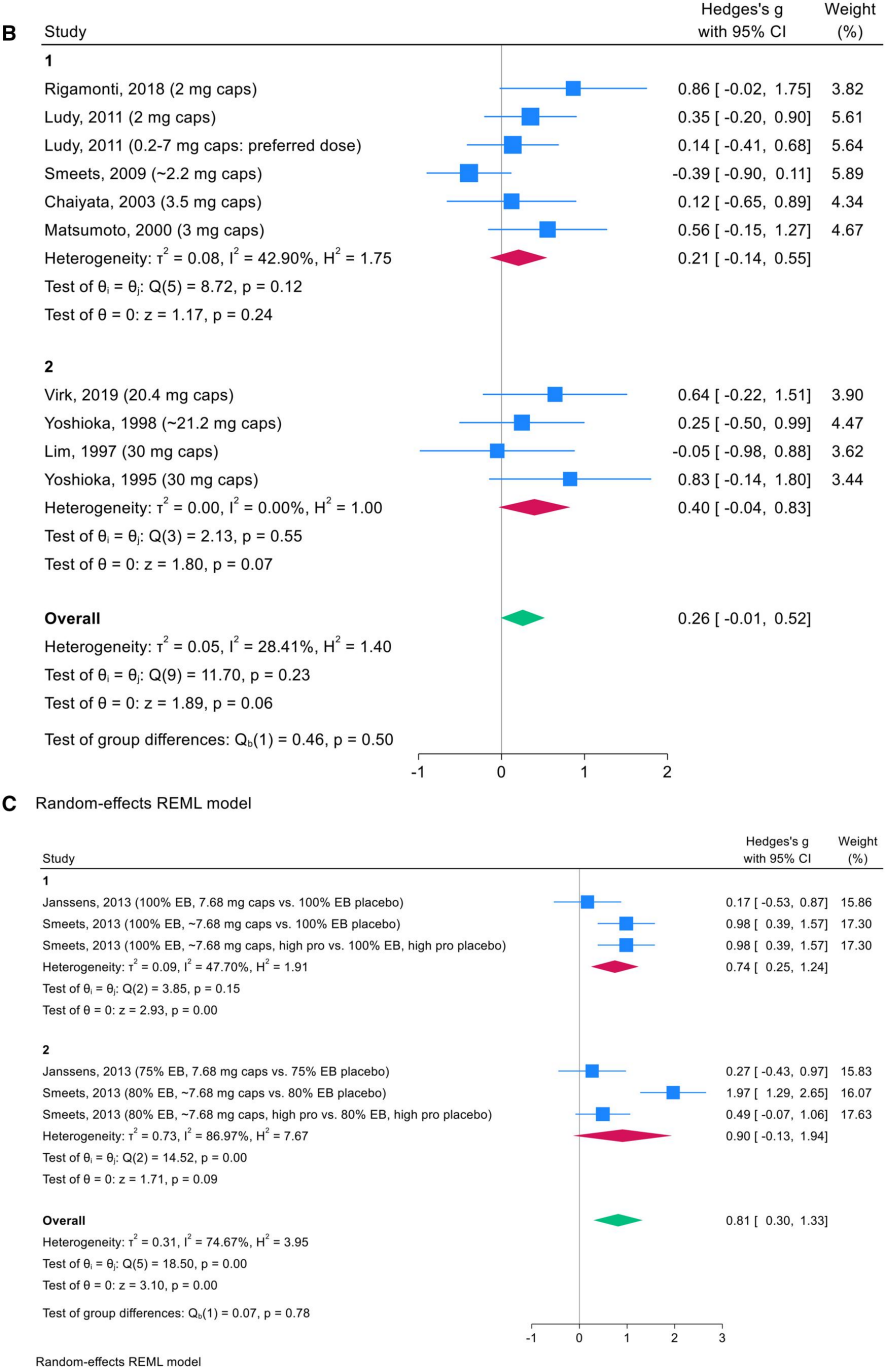

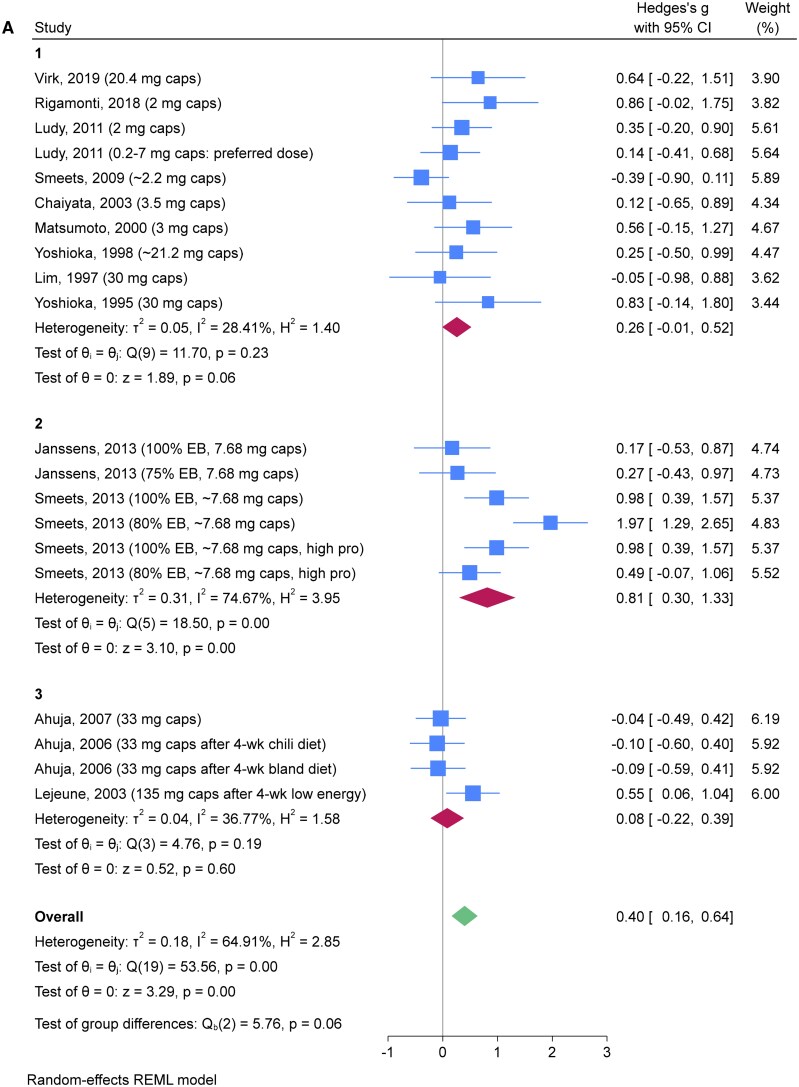
Forest Plots Comparing Studies on the Effects of Capsaicin on Energy Expenditure (EE) Using a Random-Effects REML Meta-analysis Model The weight that the studies received in the overall association is indicated by the size of the blue squares. The standardized mean difference (SMD) was used as an effect size indicator. The 95% CIs are represented by error bars. The pooled estimates for each analysis are represented by diamonds, with red diamonds representing the CI for each level of dose and green diamonds representing CI for the overall effect size. (A) Comparison by duration: 1-meal (1), 24-hour (2), and long term (3). The SMDs (95% CIs) show that capsaicin increases energy expenditure (EE) (indicated by an SMD >0) overall and when consumed over 24 hours. Capsaicin did not significantly increase EE (indicated by SMD overlapping 0) when consumed at 1-meal or over a long-term duration. (B) Comparison of 1-meal studies by dose: low (1) and medium (2). The SMDs (95% CIs) show that capsaicin did not significantly increase EE (indicated by SMD overlapping 0) at a low dose, medium dose, or overall. (C) Comparison by energy balance (EB): controlled EB (1) and controlled negative EB (2). The SMDs (95% CIs) show that capsaicin increases EE (indicated by an SMD >0) overall and in controlled EB. Capsaicin did not significantly increase EE (indicated by SMD overlapping 0) in controlled negative EB. (D) Comparison by EB and protein level: normal-protein, controlled EB (1); high-protein, controlled EB (2); normal-protein, controlled negative EB (3); and high-protein, controlled negative EB (4). The SMDs (95% CIs) show that capsaicin increases EE (indicated by an SMD >0) overall and in high-protein, controlled EB. Capsaicin did not significantly increase EE (indicated by an SMD overlapping 0) in normal-protein, controlled EB; normal-protein, controlled negative EB; or high-protein, controlled negative EB. Abbreviations: caps, capsaicin; pro, protein; REML, restricted maximum likelihood.

**Figure 3.**
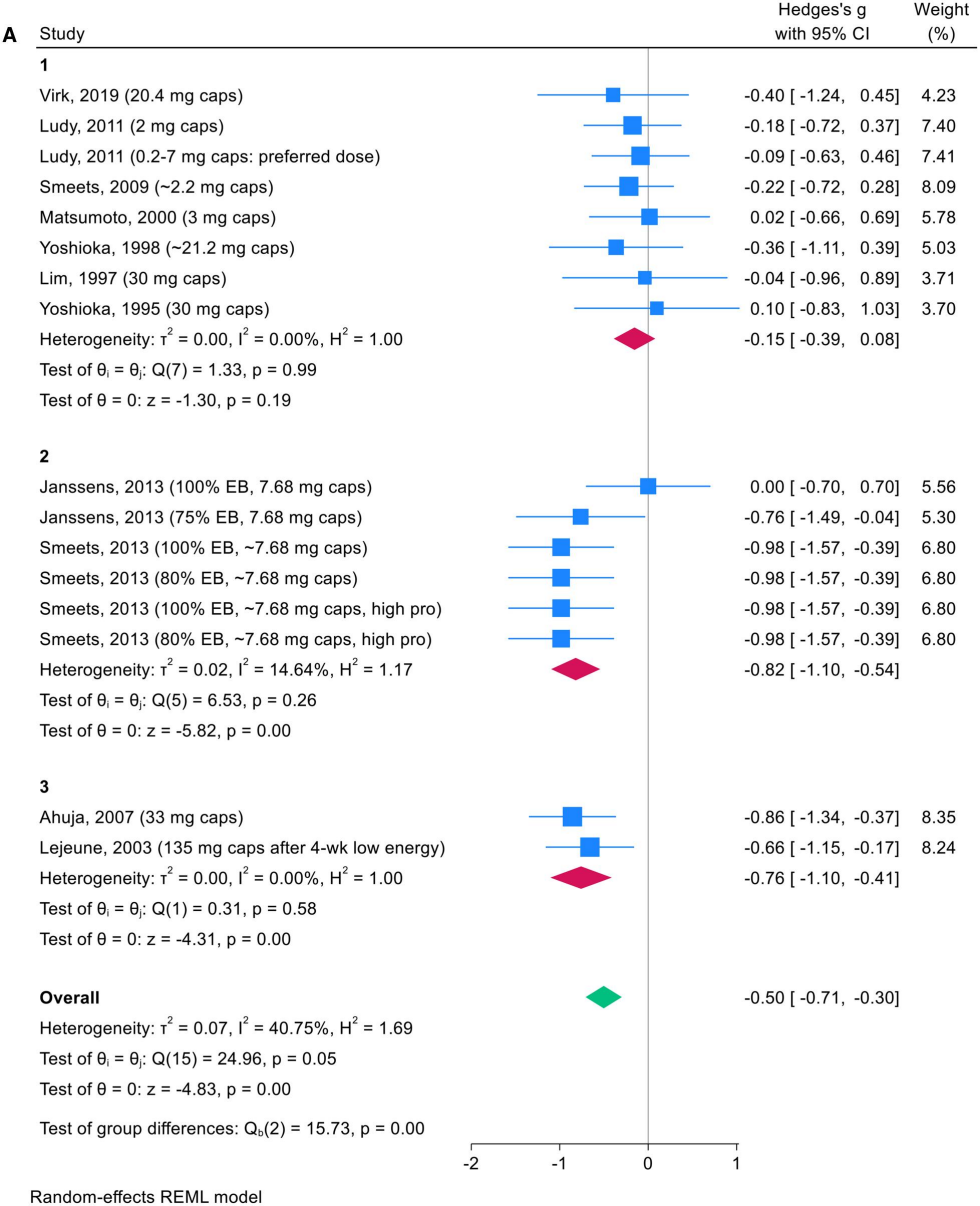

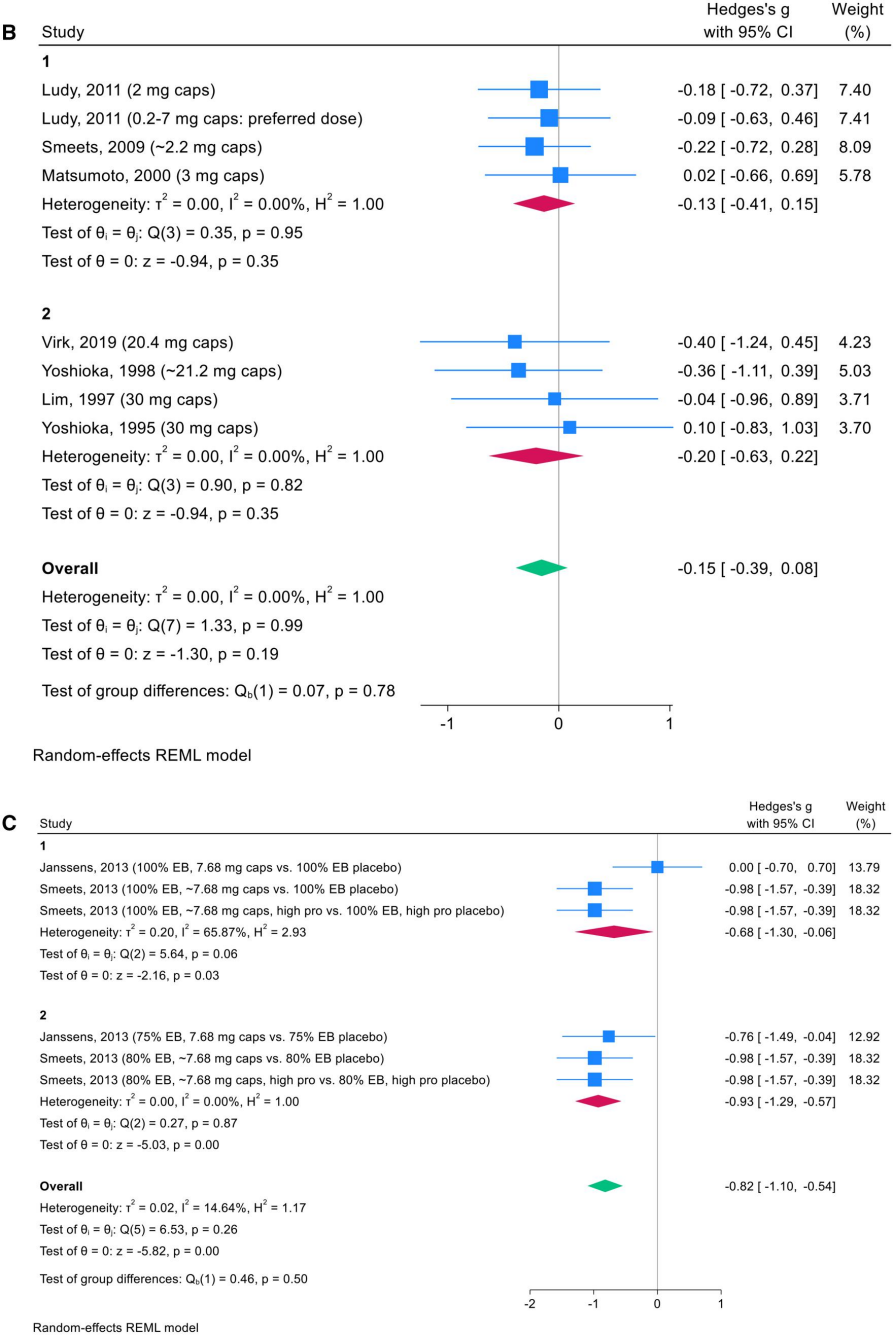

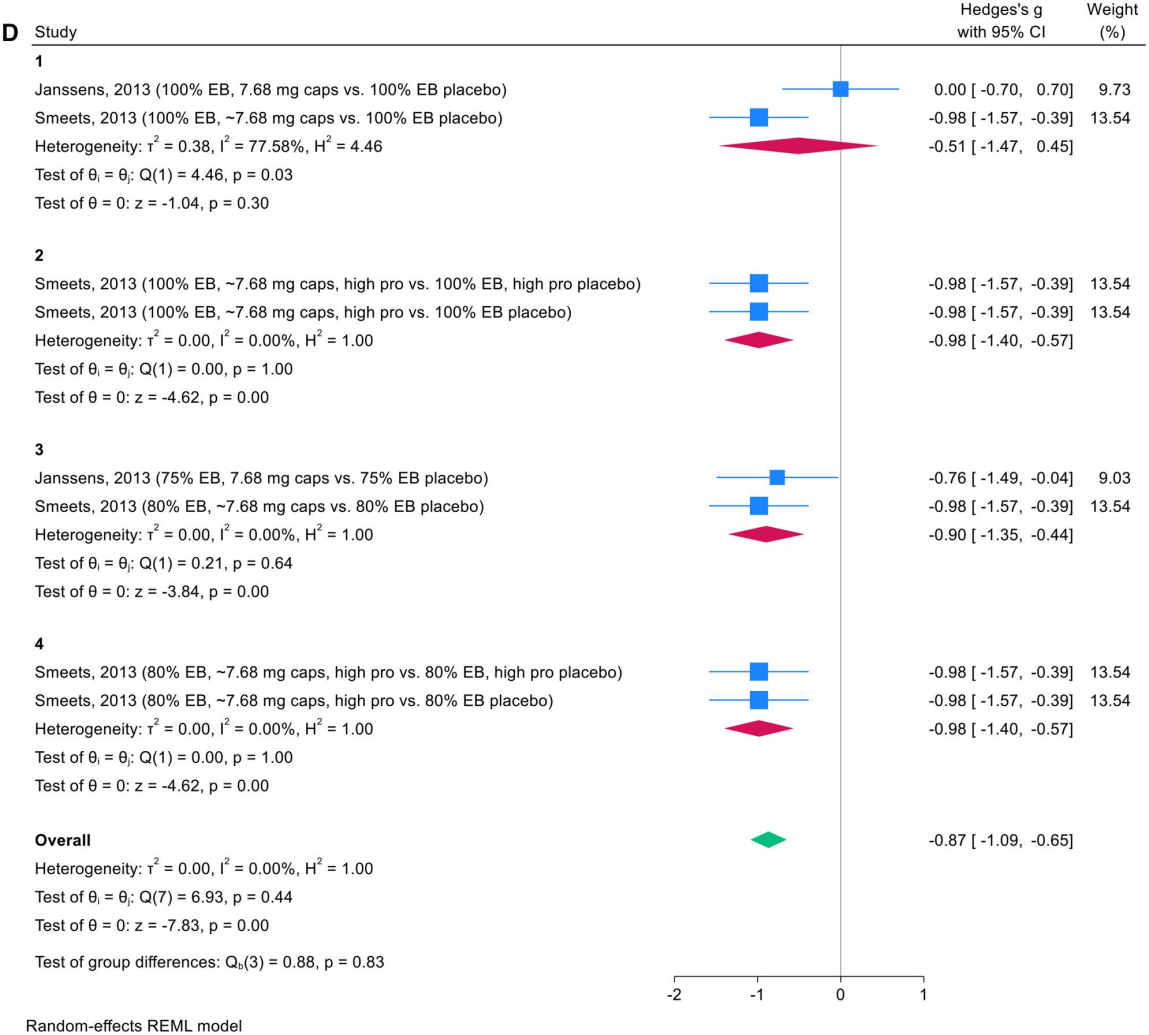
Forest Plots Comparing Studies on the Effects of Capsaicin on Substrate Oxidation (Respiratory Quotient) Using a Random-Effects REML Meta-analysis Model The weight that the studies received in the overall association is indicated by the size of the blue squares. The standardized mean difference (SMD) was used as an effect size indicator. The 95% CIs are represented by error bars. The pooled estimates for each analysis are represented by diamonds, with red diamonds representing the CI for each level of dose and green diamonds representing CI for the overall effect size. (A) Comparison by duration: 1-meal (1), 24-hour (2), and long-term (3). The SMDs (95% CIs) show that capsaicin enhances fat oxidation (indicated by an SMD <0) overall, as well as when consumed over 24 hours and long term. Capsaicin did not significantly enhance fat oxidation when consumed at 1 meal. (B) Comparison of 1-meal studies by dose: low (1) and medium (2). The SMDs (95% CIs) show that capsaicin did not significantly enhance fat oxidation (indicated by an SMD overlapping 0) at a low dose, medium dose, or overall. (C) Comparison by energy balance (EB): controlled EB (1) and controlled negative EB (2). The SMDs (95% CIs) show that capsaicin enhances fat oxidation (indicated by an SMD <0) in controlled EB, in controlled negative EB, and overall. (D) Comparison by EB and protein level: normal-protein, controlled EB (1); high-protein, controlled EB (2); normal-protein, controlled negative EB (3); and high-protein, controlled negative EB (4). The SMDs (95% CIs) show that capsaicin enhances fat oxidation (indicated by an SMD <0) overall, as well as in high-protein, controlled EB; normal-protein, controlled negative EB; and high-protein, controlled negative EB. Capsaicin did not significantly enhance fat oxidation (indicated by an SMD overlapping 0) in normal-protein, controlled EB. Abbreviations: caps, capsaicin; pro, protein; REML, restricted maximum likelihood.

Meta-analyses across all durations showed that EE was increased when capsaicin was added to the menu compared with placebo; however, this effect was driven by the 24-hour studies. In contrast, the increase in EE in 1-meal and long-term studies did not reach statistical significance (**Figure 2A**). For RQ, overall values were decreased when capsaicin was added vs placebo, primarily due to significant reductions observed in 24-hour and long-term studies, while effects on RQ in 1-meal studies did not reach statistical significance (**Figure 3A**).

### One-Meal Studies on EE and RQ

Meta-analyses of 10 1-meal studies showed that the increase in DIT did not reach statistical significance 2.5–3 hours after consuming a meal with capsaicin vs placebo (SMD = 0.26; 95% CI, –0.01 to 0.52) (**Figure 2A**). Subgroup analyses by dose also did not reach statistical significance: low dose (SMD = 0.21; 95% CI, –0.14 to 0.55) and medium dose (SMD = 0.40; 95% CI, –0.04 to 0.83) (**Figure 2B**). Similarly, RQ during DIT showed that effects did not reach statistical significance in 1-meal studies (SMD = 0.15; 95% CI, –0.39 to 0.09) (**Figure 3A**) or by dose (low: SMD = –0.13; 95% CI, –0.41 to 0.15; medium: SMD = 0.20; 95% CI, –0.63 to 0.22) (**Figure 3B**).

Although several individual studies reported significant increases in DIT with low or medium doses of capsaicin,^23^^,^^26^^,^^29–31^^,^^34^^,^^36^ others did not.^28^^,^^30^^,^^35^ These discrepancies were explained by differences in participant characteristics (eg, body weight,^30^ habitual spicy food use^26^) meal timing (lunch vs breakfast^28^), and inclusion of exercise protocols.^35^ Dose classifications do not represent experimental studies with different doses consumed by the same participants. Low-dose studies were primarily conducted in White participants and medium-dose studies in Asian participants, suggesting that most participants received a dose typical for their ethnic dietary traditions.

Taken together, 1-meal studies showed high variability. Hedges’ *g* with 95% CIs for each individual study overlapped zero, and pooled estimates showed substantial heterogeneity, reflecting inconsistency both within and between studies. Consequently, the observed increase in DIT at habitual doses did not reach statistical significance, and effects on RQ likewise did not reach significance.

### Long-Term Studies on EE and RQ

Three randomized controlled parallel-arm studies examined long-term effects on EE,^42–44^ with 2 also measuring RQ.^42^^,^^44^ One of these studies, Ahuja et al,^43^ included 2 arms: 1 following a 4-week chili diet and the other following a 4-week bland diet. Two studies applied a medium dose of capsaicin vs placebo in approximate EB,^42^^,^^43^ while 1 study applied a high dose of capsaicin vs placebo (135 mg/d, over 3 meals) during weight regain, thus in positive EB following a period of negative EB (**Figure 1**).^44^ In middle-aged participants with overweight, the increase in EE after consuming a medium dose of capsaicin vs placebo did not reach statistical significance during 4 weeks in approximate EB, with 1 or 2 meals per day.^42^^,^^43^ However, RQ was significantly decreased.^43^ In a randomized double-blind, placebo-controlled study, middle-aged participants with overweight or obesity received a high dose of capsaicin vs placebo during a 3-month weight-maintenance period after a 4-week loss of 6.6 ± 2.0 kg (7.8% ± 1.8% of initial body mass).^44^ While weight regain occurred similarly in the capsaicin and placebo groups during follow-up, RQ was significantly less increased in the treatment group compared with placebo, indicating a relatively more sustained higher fat oxidation.

Meta-analyses showed that the increase in EE after capsaicin vs placebo over the long term did not reach statistical significance (SMD = 0.08; 95% CI, –0.22 to 0.39) (**Figure 2A**), but there was a statistically significant reduction in RQ after long-term consumption of a medium or high dose of capsaicin vs placebo (SMD = –0.76; 95% CI, –1.10 to –0.41) (**Figure 3A**). The high-dose study^44^ was excluded from meta-analysis due to differences in EB context (positive EB during weight regain) and dose delivery (supplement capsules). Overall, long-term capsaicin consumption in free-living conditions did not provide statistically significant evidence for an EE increase but did significantly reduce RQ, suggesting sustained higher fat oxidation.

In summary, across 1-meal and long-term studies, variability was high due to differences in participant characteristics, study protocols, and habitual spicy food intake. The increase in EE did not reach statistical significance, whereas the significant reduction in RQ following long-term consumption of a medium dose of capsaicin suggests a sustained effect on fat oxidation.

### 24-Hour Studies on EE and RQ

Two randomized controlled 24-hour crossover studies^38^^,^^39^ were included in the present meta-analysis. One of these studies, Smeets et al,^39^ included 2 arms: one with capsaicin alone and the other combining capsaicin with high protein. Subgroup analysis by EB showed that capsaicin significantly increased EE in controlled EB (SMD = 0.74; 95% CI, 0.25 to 1.24), while the increase in EE during controlled negative EB did not reach statistical significance (SMD = 0.90; 95% CI, –0.13 to 1.94) (**Figure 2C**). When further analyzed by protein level, capsaicin significantly increased EE only in the high-protein, controlled EB condition (SMD = 1.70; 95% CI, 0.26 to 3.15). In other conditions, including controlled EB (SMD = 0.60; 95% CI, –0.20 to 1.40), controlled negative EB (SMD = 1.12; 95% CI, –0.55 to 2.78), or high-protein, controlled negative EB (SMD = 1.95; 95% CI, –0.94 to 4.84) (**Figure 2D**), the increase in EE did not reach statistical significance.

For RQ, subgroup analysis by EB showed that capsaicin significantly decreased RQ in both controlled EB (SMD = –0.68; 95% CI, –1.30 to –0.06) and controlled negative EB (SMD = –0.93; 95% CI, –1.29 to –0.57) (**Figure 3C**). When further analyzed by protein level, capsaicin significantly decreased RQ in high-protein controlled EB (SMD = –0.98; 95% CI, –1.40 to –0.57), controlled negative EB (SMD = –0.90; 95% CI, –1.35 to –0.44), and high-protein, controlled negative EB (SMD = –0.98; 95% CI, –1.40 to –0.57) conditions, but not in controlled EB (SMD = –0.51; 95% CI, –1.47 to 0.45) (**Figure 3D**).

In summary, 24-hour controlled studies suggest that capsaicin combined with protein can increase EE in controlled EB, and capsaicin consumption consistently enhances fat oxidation in negative EB or when combined with high protein intake.

### One-Meal Studies on Appetite and EI

All 10 studies on appetite and EI-related parameters were conducted in approximate EB.^23–28^^,^^32^^,^^33^ Seven administered a low dose of capsaicin vs placebo,^23–28^ while 3 studies administered a medium dose^32^^,^^33^ (**Figure 1**).

Energy intake did not differ between capsaicin and placebo conditions in young Japanese female participants with normal weight after consumption of a medium dose at breakfast,^33^ in young Japanese men with normal weight after consumption of a low-to-medium dose,^32^ in young men and women with normal weight who liked hot spices after a low dose in a starter meal,^27^ or in young lean men and women at a meal challenge 4.5 hours after ingesting a low dose.^25^ Except for 1 study,^33^ these trials showed that differences in visual analog scale (VAS) ratings for hunger, desire to eat, satiety, or fullness did not reach statistical significance between conditions.

However, EI was reduced in young White men with normal weight^33^ after ingesting a medium dose as an appetizer. Additionally, in nonusers vs users with similar characteristics, EI was reduced in nonusers during a 4.5-hour period after a meal with a low dose, paralleled by reductions in hunger and desire-to-eat VAS ratings.^26^ After a low dose, differences in EI, VAS ratings, or circulating concentrations of ghrelin, GLP-1, or PYY did not reach statistical significance during the 3 hours immediately after food ingestion by young men and women with obesity.^23^ Also, in young participants with normal weight or overweight, differences in satiety and PYY did not reach statistical significance, but GLP-1 was significantly increased after a lunch containing a low dose of capsaicin.^28^ Increased satiety was observed in young men and women with normal weight when capsaicin was administered by intraduodenal infusion.^24^ This did not affect plasma concentrations of GLP-1 or PYY, but satiety appeared to be related to gastrointestinal stress, as shown by associations with pain, burning sensation, nausea, and bloating scores.^24^ Taken together, most effects of capsaicin vs placebo during and after 1-meal studies on hunger, satiety, desire to eat, fullness, EI, and satiety hormones did not reach statistical significance, with some exceptions.

### 24-Hour Studies on Appetite and EI

Two of the four 24-hour studies on appetite and EI-related parameters included 1 study in approximate EB^41^ and 1 study conducted in both in negative and positive EB.^40^ All studies provided a low dose of capsaicin. Ad libitum EI showed a 10%–12% reduction with capsaicin consumption vs placebo in capsules, and a 16% reduction when ingested in tomato juice. Increased satiety paralleled these observations over 2 consecutive days in young men and women with normal weight, overweight, or obesity.^41^

A 24-hour study after 3 weeks of negative and 3 weeks of positive EB in young men and women who consumed a low dose of capsaicin vs placebo showed that capsaicin suppressed hunger and increased satiety during both negative and positive EB.^40^ Two additional studies were conducted in strictly controlled EB.^37^^,^^39^ Fullness and satiety appeared to be higher with a low dose vs placebo, while differences in desire to eat, satiety, and fullness between 25% negative EB with capsaicin and EB with placebo in young men and women with normal weight to overweight did not reach statistical significance. Differences in ad libitum EI also did not reach statistical significance between conditions in EB or negative EB.^37^

Subsequently, the impact of protein and capsaicin, alone or combined, on fullness during 20% EI restriction and during controlled EB was investigated in young men and women with normal weight or overweight.^39^ In 20% negative EB, fullness was decreased in the control condition but not in the conditions with a low dose of capsaicin, elevated protein, or both. It was concluded that protein and capsaicin, consumed alone or in combination, counteracted the EI restriction effects on fullness.^39^

Taken together, the 24-hour controlled studies showed increased fullness and satiety whether conducted in EB or negative EB, and incidental decreases in EI with habitual-dose capsaicin vs placebo. In negative EB, protein and capsaicin, consumed alone or mixed, counteracted the EI restriction effects on fullness. Most other differences in appetite ratings did not reach statistical significance.

### Long-Term Studies on Appetite and EI

Two randomized controlled, parallel-arm, long-term studies were identified.^42^^,^^44^ In a randomized crossover study involving 4 weeks of low-dose capsaicin exposure and 4 weeks of placebo, differences in EI did not reach statistical significance in middle-aged women with overweight or obesity who were infrequent users.^42^ Another parallel-arm study involved men and women with overweight and obesity who were tested after a 4-week weight-loss period followed by a 3-month weight-maintenance period. Forty-two participants consumed a high dose of capsaicin in capsules during weight maintenance, while 49 participants consumed placebo capsules. Differences in hunger and satiety ratings between the 2 groups did not reach statistical significance during weight maintenance.^44^

In summary, studies examining appetite and EI following ingestion of a habitual dose of capsaicin vs placebo consumed before or during a single meal generally did not reach statistical significance for appetite or meal size. This lack of statistically significant effect was also observed with medium or high doses during long-term, uncontrolled field studies. However, when capsaicin was consumed before or during each meal over the course of at least a full day in EB, fullness and satiety were increased and EI was incidentally reduced. Although effects were small, protein and capsaicin, consumed alone or combined, counteracted the effects of EI restriction on fullness in negative EB.

## DISCUSSION

Red pepper, containing capsaicin as its pungent principle, consumed in culinary doses vs placebo increases total daily EE, fullness, and satiety across sex (men, women) and body weight (normal weight, overweight, obesity) categories, but only when consumed throughout the full day with each meal. The effects on EE were primarily demonstrated by meta-analyses of controlled 24-hour studies in both EB and negative EB conditions. The effects on fullness and satiety, and incidentally on EI, as demonstrated in the 24-hour studies, appeared to increase over the course of the day, as capsaicin consumption was successively repeated.

When offered with just 1 meal, in a dosage representative of habitual use, the increase in DIT did not reach statistical significance, as shown by meta-analyses. This was likely due to high variation in participant characteristics and study design, both between and within studies. Similarly, differences in appetite and EI generally did not reach statistical significance in 1-meal studies. Regarding mechanisms explaining possible effects on appetite, no consistent differences in circulating concentrations of ghrelin, GLP-1, and PYY, nor consistent associations with VAS satiety scores, were observed after a low dose of capsaicin vs placebo in 3 different studies.^23^^,^^24^^,^^28^ The effect on satiety appeared to be more related to gastrointestinal stress, as indicated by associations with pain, burning sensation, nausea, and bloating scores.^24^ Previously, a possible satiety effect was partly ascribed to taste receptor activation.^45–53^ Recently, Cunningham et al^55^ showed that an increase in spiciness resulted in an 18% reduction in food intake and a 17% reduction in eating rate. In fact, we were not able to test different doses systematically, since most participants received a usual dose for their ethnicity. A few studies applied different doses in the same participants, and these reported greater decreases in EI at higher doses, especially in nonusers.^26^^,^^32^

Over the long term, in free-living conditions, meta-analyses did not provide statistically significant evidence for an EE change in capsaicin vs placebo, apart from a small effect observed after 3 months: EE decreased less in the capsaicin condition than in the placebo condition. Importantly, EE did not differ from baseline EE, suggesting a contribution to preventing adaptive thermogenesis.^44^ The weak effect of capsaicin vs placebo on EE after 3 months of use may be due to large variations in participant characteristics (eg, sex, ethnicity, body weight, and frequency of RP use), as seen in 1-meal studies. Energy intake was not measured during the free-living long-term studies. The dose of capsaicin in the study by Lejeune et al^44^ was based on the highest dose used in the Yoshioka studies^33–36^ and was administered to White participants 3 times per day (45 mg before each meal). This dose likely was too high for the participants and may have limited compliance.^44^

Despite the statistically significant increases in EE and fullness observed in the meta-analyses of the 24-hour controlled studies, the magnitudes of those increases were small. However, they were sufficient to counteract negative EB effects, such as adaptive thermogenesis, increased hunger, and decreased fullness during 20%–25% negative EB. Elevated protein intake combined with capsaicin consumption further mitigated these negative EB effects. Counteracting effects of decreased fullness, satiety, and adaptive thermogenesis have been demonstrated as proof-of-principle in meta-analyses of controlled 24-hour studies.^5^^,^^7–14^^,^^56–61^ This may contribute to body-weight maintenance after body-weight loss.

Although RQ, an index of substrate oxidation, did not differ significantly between capsaicin and placebo conditions during 1-meal studies in approximate EB, RQ was significantly reduced during the 24-hour studies in EB as well as in the negative EB condition with capsaicin consumption compared with placebo (**Figure 3C**). This suggests that fat oxidation was substantively greater with capsaicin, at the cost of carbohydrate oxidation. Combining capsaicin with protein resulted in a lower RQ and a more negative fat balance in both EB and negative EB conditions (**Figure 3D**). Additionally, the significantly lower RQ observed in meta-analyses of long-term studies indicates higher fat oxidation, which may contribute favorably to body composition during weight regain.

### Strengths and Limitations

The present review and meta-analysis has some strengths and limitations. The collection of, in total, 24 studies on EE, RQ, and/or EI and appetite effects of capsaicin vs placebo during 1 meal, 24 hours, or over the long term can be characterized as a strength. At the same time, the variety in participant characteristics and study design may be regarded as a limitation. This is partly addressed by including fully controlled 24-hour studies, which show proof-of-principle. However, the observed effects are small and do not consistently appear in 1-meal or long-term studies with different designs. Another limitation is the lack of the possibility to study dose effects. Distinguishing low and medium doses appeared not to be an applicable strategy, since most participants received usual doses. The possibility to study a duration effect allowed us to show that some effects, especially those on fullness, satiety, and EI, are additive over all meals during a day. Finally, the small number of long-term studies with completely different designs may be regarded as a limitation, although these studies allowed a convincing long-term observation of decreased RQ—that is, higher fat oxidation.

## FUTURE DIRECTIONS

Challenges to weight-loss maintenance, such as increased hunger, desire to eat, loss of FFM (the main determinant of EE), and reductions in EE and fat oxidation, persist over time. Additionally, large variations in participant characteristics and limited compliance can result in misclassification of responders vs nonresponders to a given treatment. Therefore, an individualized approach to weight management is warranted.

The heterogeneity in response to behavioral obesity treatment, influenced by environmental, social, and biological factors, supports the need for intervention science to move toward a precision medicine approach that matches individuals to their most suitable obesity treatments.^60–71^ Different responses have been observed regarding eating behavior and changes therein,^60^ as well as body composition, EE, and fat oxidation.^61^^,^^63^^,^^64^ A precision nutrition and precision medicine approach to long-term weight maintenance should be multifactorial, including behavioral counseling and lifestyle interventions such as increased physical activity and circadian alignment.^60–71^

We speculate that, alongside pharmacotherapy, consuming food ingredients such as RP and protein may provide individuals with obesity with a range of potentially effective treatment options that can be tailored to their needs and preferences.^60–72^ Pharmacotherapy with a GLP-1 analog such as tirzepatide moderates hunger and desire to eat and reduces the reward value of food ingestion, contributing to decreased EI.^73^^,^^74^ Tirzepatide also stimulates fat oxidation, thereby increasing fat loss and sparing FFM.^74^ It enhances fat utilization while reducing carbohydrate and protein utilization, playing an important role in energy metabolism.^74^ However, EE following tirzepatide treatment failed to show sustained EE or prevent adaptive thermogenesis. The counterregulatory action of tirzepatide on metabolic adaptation diminished as weight loss slowed.^74^

We suggest that capsaicin, particularly when combined with protein, may serve as a useful addition to other treatments. Since capsaicin and protein consumption, alone or combined, vs placebo was shown to counteract decreased fullness and adaptive thermogenesis during negative EB and promote fat oxidation over the long term, capsaicin may be a valuable ally in efforts to moderate trends in body weight.

## Data Availability

The data supporting this work are derived from previously published sources cited in the manuscript. The compiled extraction and analysis spreadsheet is available from the corresponding author on reasonable request.
